# P-2144. *Candida auris* skin colonization and the risk of subsequent *C. auris* bloodstream infection in two teaching hospitals

**DOI:** 10.1093/ofid/ofae631.2298

**Published:** 2025-01-29

**Authors:** Polyxeni Karakosta, Paraskevi Mantzana, Maria Siopi, Georgios Meletis, Areti Tychala, Panagiota Christina Georgiou, Efthymia Protonotariou, Iosif Meletiadis, Lemonia Skoura, Spyros Pournaras

**Affiliations:** Attikon University General Hospital, Medical School, National and Kapodistrian University of Athens, Athens, Greece, Athens, Attiki, Greece; Ahepa University Hospital, Thessaloniki, Thessaloniki, Greece; NKUA, Athens, Attiki, Greece; AHEPA University Hospital, Medical School, Faculty of Health Sciences, Aristotle University of Thessaloniki, Thessaloniki, Greece., Thessaloniki, Thessaloniki, Greece; AHEPA Hospital, Thessaloniki, Thessaloniki, Greece; Attikon University General Hospital, Medical School, National and Kapodistrian University of Athens, Athens, Greece, Athens, Attiki, Greece; AHEPA University Hospital, Medical School, Faculty of Health Sciences, Aristotle University of Thessaloniki, Thessaloniki, Greece., Thessaloniki, Thessaloniki, Greece; NKUA, Athens, Attiki, Greece; AHEPA University Hospital, Medical School, Faculty of Health Sciences, Aristotle University of Thessaloniki, Thessaloniki, Greece., Thessaloniki, Thessaloniki, Greece; Attikon University General Hospital, Medical School, National and Kapodistrian University of Athens, Athens, Attiki, Greece

## Abstract

**Background:**

*Candida auris*, an emerging yeast that can cause outbreaks of severe infections in healthcare facilities, has shown an increased spread since the start of the COVID-19 pandemic. This study aimed to investigate the relative risk of subsequent *C. auris* bloodstream infection (BSI) in patients colonized on their skin by *C. auris*.

**Figure:**
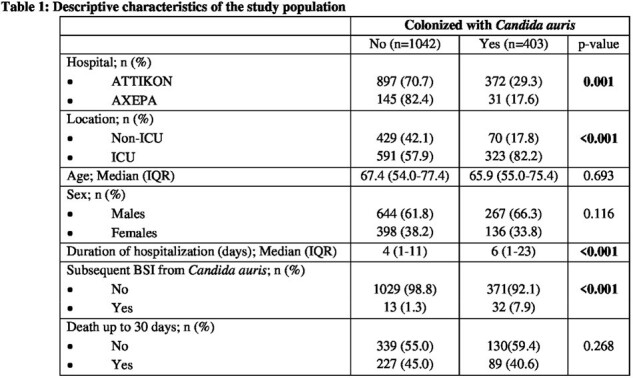

**Methods:**

In this retrospective study, we included all inpatients hospitalized from January 2021 to December 2023, in Intensive Care Units (ICUs) or internal medicine wards of two distant teaching hospitals (Athens: Attikon University Hospital, Thessaloniki: AHEPA University Hospital), who were screened for skin colonization by *C. auris* and did not have a history of infection or colonisation by *C. auris*. The axilla and groin served as sampling sites. Screening was performed by selective media and species identification by MALDI-TOF. Patients were followed up for *C. auris* BSI and relative risk (95% confidence interval) was estimated after adjustment for ICU hospitalization, duration of hospitalization, age and sex.

**Figure:**
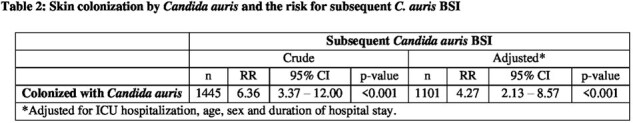

**Results:**

Of 1445 patients screened, 403 (27.9%) were colonized by *C. auris* (17.8% non-ICU, 82.2% ICU patients). Among them, 45 (3.1%) subsequently developed *C. auris* BSI. Skin *C. auris* colonization was associated with more than a 4-fold higher risk of subsequent *C. auris* BSI, after adjusted for ICU hospitalization, age, sex and duration of hospital stay [RR (95%CI): 4.27 (2.13-8.57)].

**Conclusion:**

*C. auris* skin colonization increases significantly the risk of developing *C. auris* BSI. This underscores the significance of public health monitoring and containment strategies for *C. auris*. Adhering to infection control protocols is crucial, and healthcare providers should take this into account when deciding on initial treatment, although more comprehensive genome-wide data are necessary to confirm horizontal transmission.

**Disclosures:**

All Authors: No reported disclosures

